# WASHC3 knockout disrupts mitochondrial protein homeostasis and energy metabolism in cardiomyocytes

**DOI:** 10.3389/fcvm.2026.1682381

**Published:** 2026-02-12

**Authors:** Sujin Kim, Deung-Dae Park, Amelia Glazier, Wolfgang Rottbauer, Steffen Just

**Affiliations:** 1 Molecular Cardiology, Department of Internal Medicine II, University of Ulm, Ulm, Germany; 2 Department of Internal Medicine II, University of Ulm, Ulm, Germany

**Keywords:** mitochondrial function, proteomics, WASH complex, WASHC3, zebrafish

## Abstract

**Introduction:**

The WASH complex regulates endosomal actin dynamics and vesicular trafficking and is essential for neuronal integrity and motor function. Although variants in WASHC4, WASHC5, and WASHC3 are linked to neurodevelopmental abnormalities, the role of WASHC3 beyond the nervous system, particularly in cardiac mitochondrial regulation, remains unclear.

**Methods:**

We modeled WASHC3 loss of function in zebrafish and human cardiomyocytes. Washc3 was suppressed in zebrafish embryos by antisense oligonucleotide-mediated knockdown, and a stable Washc3 knockout line was generated using CRISPR/Cas9. Washc3-deficient zebrafish hearts were analyzed by quantitative LC-MS/MS proteomics with GO/KEGG enrichment and transcript-level assays. Mitochondrial bioenergetics was assessed by Seahorse XF assays in primary zebrafish cardiomyocytes and in human AC16 cardiomyocytes following AAV-shRNA-mediated WASHC3 knockdown.

**Results:**

Washc3 knockdown embryos exhibited neuromuscular degeneration, impaired locomotion, and early cardiac dysfunction. In contrast, Washc3 knockout zebrafish showed normal early development but developed progressive pericardial degeneration and epicardial remodeling in aged animals. Cardiac proteomics revealed downregulation of mitochondrial proteins, particularly oxidative phosphorylation components, supported by pathway enrichment and concordant transcript-level findings. Mitochondrial respiration was significantly impaired in both Washc3-deficient zebrafish cardiomyocytes and WASHC3-depleted human AC16 cardiomyocytes.

**Discussion:**

These findings identify a previously unrecognized role for WASHC3 in maintaining cardiac mitochondrial protein homeostasis and bioenergetic function and provide a framework linking neuromuscular and cardiac phenotypes to impaired mitochondrial bioenergetics in energy-demanding tissues.

## Introduction

Striated muscles, comprising both cardiac and skeletal muscle tissue, are essential for maintaining vital physiological functions such as circulatory homeostasis and locomotion. As highly specialized contractile systems, their proper development and maintenance are critical for organismal survival (1). Recent studies have implicated the Wiskott-Aldrich syndrome protein and SCAR homologue (WASH) complex in the regulation of striated muscle integrity (2). The WASH complex is a heteropentameric assembly consisting of five subunits: WASHC1 (WASH1), WASHC2 (FAM21), WASHC3 (CCDC53), WASHC4 (SWIP), and WASHC5 (Strumpellin) (3). Clinical studies have implicated mutations in components of the WASH complex—particularly WASHC3 (4), WASHC4 (5, 6), and WASHC5 (7, 8)—in a spectrum of developmental disorders and skeletal muscle pathologies. *In vivo* studies further support a role for the WASH complex in muscle physiology. Knockdown of *washc5* in zebrafish results in reduced motility and impaired cardiac contractility (9), and heterozygous *washc5*-deficient zebrafish and mice exhibit adult-onset cardiac dysfunction, while homozygous *washc5* knockout results in embryonic lethality due to cardiac failure (10). Similarly, *washc4* knockdown in zebrafish embryos leads to progressive structural and functional deficits in both cardiac and skeletal muscle (2). Together, these findings indicate an important underexplored role of the WASH complex in maintaining striated muscle homeostasis and suggest that mutations in individual WASH subunits might contribute not only to neurodevelopmental disorders but also to primary myopathies and cardiomyopathies.

Mitochondrial respiration is fundamental for fulfilling the high energetic demands of cardiac and skeletal muscle and for sustaining their physiological function. In the heart, the majority of ATP is produced via mitochondrial oxidative phosphorylation (OXPHOS) (11). Disruptions in mitochondrial ATP synthesis are strongly implicated in the pathogenesis of cardiomyopathies. Such impairments can arise through multiple mechanisms, including dysfunction of electron transport chain (ETC) complexes or their subunits, mutations in mitochondrial or nuclear genes encoding mitochondrial proteins, defects in mitochondrial protein import systems (12, 13), or endosomal dysfunctions (14, 15). These findings underscore the critical importance of maintaining mitochondrial homeostasis—from biogenesis to degradation—for preserving both mitochondrial integrity and efficient energy production in muscle tissue (16, 17). Accordingly, elucidating the regulatory axes that govern mitochondrial function—either directly or through indirect mechanisms—remains a critical area of investigation.

Although the pathogenic consequences of disrupting various WASH complex subunits have been previously investigated (5, 8), the specific function and physiological relevance of WASHC3 remain largely undefined. Previous studies have primarily linked WASHC3 deficiency to neurodevelopmental abnormalities in human patients (4). However, the underlying molecular and cellular mechanisms remain largely unresolved, and potential roles of WASHC3 beyond the nervous system have not been addressed. Based on our own preliminary data, which suggested additional effects on cardiac function, we aimed to elucidate the mechanistic basis of the observed phenotypes, with a particular focus on cardiac biology and mitochondrial regulation. In this study, we indeed identify a role for Washc3, in addition to its function during neurodevelopment, in zebrafish cardiac and skeletal muscle development and homeostasis. Both morpholino-mediated knockdown and CRISPR/Cas9-based knockout of *washc3* in zebrafish embryos resulted in morphological abnormalities affecting striated muscle development. In aged zebrafish, *washc3*-deficient animals exhibited cardiac malformations, including degeneration of the pericardial membrane and pronounced epicardial thickening. Proteomic profiling of Washc3 mutant zebrafish hearts revealed a significant downregulation of mitochondrial proteins, particularly those involved in the electron transport chain, which is essential for ATP synthesis. Notably, these molecular changes occurred in the absence of overt ultrastructural alterations in mitochondrial morphology. Functional impairment of mitochondrial respiration was confirmed by Seahorse/extracellular flux (XF) assays using both primary cardiomyocytes from Washc3 mutant zebrafish and WASHC3-depleted human cardiomyocytes, suggesting a conserved role for WASHC3 in mitochondrial function. Together, these findings uncover a previously unrecognized role of Washc3 in maintaining mitochondrial proteostasis and bioenergetic capacity in the heart, highlighting its importance for cardiac integrity and function.

## Materials and methods

### Experimental zebrafish and maintenance

This study was conducted under institutional approvals [Regierungspräsidium Tübingen No.1627; Tierforschungszentrum (TFZ) Ulm University No. z.183], and in compliance with EU Directive 2010/63/EU. All experiments were performed in accordance with the ARRIVE guidelines and relevant institutional regulations. Zebrafish (Danio rerio) care and breeding were carried out as previously described (18).

### Microinjection and *washc3*-knockout zebrafish line generation by CRISPR/Cas9 system

Morpholino-modified antisense oligonucleotides (MO) targeting the splice doner site of the exon2-intron 2 was designed (Gene Tools, LLC, Oregon, USA), while a Standard Control MO (Ctrl MO) was used at the same concentration as a negative control (MO-*washc3*: 5′-TGACTGTAAGATGAACAAACCTCCT-3′, Ctrl MO: 5′-CCTCTTACCTCAGTTACAATTTATA-3′). Morpholino doses (250 μM and 500 μM) were selected to assess dose dependency while avoiding overt non-specific toxicity and were subsequently referred to as MO-low and MO-high, respectively. CRISPR/Cas9 technology was employed to generate *washc3* knockout lines, with 400 ng/µL recombinant Cas9 protein (Euphoria GmbH, Germany) co-injected alongside 130 ng/µL a synthetic trans-activating crRNA (tracrRNA: AAACAGCAUAGCAAGUUAAAAUAAGGCUAGUCCGUUAUCAACUUGAAAAAGUGGCACCGAGUCGGUGCU) (Eurofins Genomics, Germany) and gene-specific crRNA (UCUACUCCUGAUCCCACAAUGUUUUAGAGCUAUGCUG UUUUG) (Eurofins Genomics, Germany) targeting *washc3* exon 1, diluted in 200 mM KCl resulted *washc3*-deficient line. Unless otherwise specified, MO injections were carried out using the TüAB wildtype strain and, microinjections were performed at the one-cell stage of fertilized zebrafish oocytes. Both morpholino-mediated knockdown and CRISPR/Cas9-mediated knockout were used to compare early developmental requirements with post-developmental loss-of-function outcomes.

### Western blot assays

Protein extractions for immunoblotting were gained from 72 hpf embryo, 1-year-old adult zebrafish heart, and shRNA transduced AC16 cell, respectively. Collected embryos (∼50) were deyolked and washed before homogenization with pestles, and TNN lysis buffer used for protein extraction form embryo. For adult heart and AC16 cell, we used RIPA buffer containing protease and phosphatase inhibitor. After incubation on ice for 1 h, all samples were centrifuged at 18,000 g for 15 min in 4 °C. Bradford assay was used for measuring protein concentration. 10–15 µg of protein in 5× Laemmli buffer were boiled for denaturation and samples loaded on precast 8%–16% SDS gels (BIO-RAD, Hercules, CA, USA) for separation. Proteins were transferred on polyvinylidene difluoride (PVDF) membranes using the Trans-Blot Turbo System (BIO-RAD, USA). Membranes were blocked in SuperBlock Blocking Buffer (Thermo Scientific, Waltham, MA, USA) for 30 min at room temperature (RT). Membranes were incubated in diluted (1:1,000) primary antibody with blocking buffer at 4 °C overnight (ON). After washing with TBST (Tris-buffered saline, 0.05% Tween 20) for 1 h at RT, membranes were incubated with the corresponding anti-rabbit or ant-mouse secondary antibody conjugated to HRP (dilution 1:5,000 in SuperBlock) for 1 h at RT and washed again with TBST for an hour. Membranes were developed using Pierce ECL Western Blotting Substrate (Thermo Fisher Scientific, Waltham, MA, USA) and a luminescent image analyzer (LI-COR Biosciences GmbH, Germany). Anti-WASHC3/CCDC53 (Rabbit; Cat# 24445-1-AP, Proteintech, IL, USA), Anti-*α*-Tubulin (mouse, Cat# GTX628802, GeneTex, CA, USA). All replicates used for the quantification were from seven independent experiments.

### Immunofluorescence staining

The whole mount immunofluorescence of muscle tissue in embryo, we adapted a published protocol (19). Briefly, embryos were treated with 0.003% PTU (1-phenyl-2-thriourea) in E3 media before 24 hpf to inhibit pigmentation and changed every day until the termination of experiment. Collected embryos were fixed in 4% paraformaldehyde (PFA) for overnight at 4 °C and washed with PBSTw (PBS + 0.1% Tween-20). Embryos incubated for 5 min in 150 mM Tris-HCL pH 9.0 at RT and heated at 70 °C for 15 min. For permeabilization, we incubated embryos with 0.1 μM/mL Proteinase K in PBSTw for 50 min and fixed again with 4% PFA at RT. All washing step in between for permeabilization performed with PBSTw. Embryos were incubated in Blocking buffer (10% sheep serum, 0.8% Triton X-100% and 1% BSA in PBSTw) for 4 h at 4 °C on a rocking platform. Primary and secondary antibody diluted in Buffer I (1% sheep serum, 0.8% Triton X-100% and 1% BSA in PBSTw) for 48 h at 4 °C and washed with PBST-TS (10% sheep serum, 1% Triton X-100 in PBS) before and after primary and secondary antibody incubation. DAPI was applied with secondary antibody and visualized using confocal microscopy. Anti-Tropomyosin (CH1, Developmental Studies Hybridoma Bank, Iowa City, IA, USA), Alexa 488 goat anti-mouse IgG2 (Cat# A-21121, Invitrogen, Waltham, MA, USA). Images were acquired using Confocal Microscopy.

Immunostaining of MF20 (MF20 concentrate, Developmental Studies Hybridoma Bank, Iowa City, IA, USA) and S46 (S46 concentrate, Developmental Studies Hybridoma Bank, Iowa City, IA, USA) was performed fixed embryos with Dent's Fixative (20% DMSO in MeOH) for overnight at RT. Next day Dent's Fixative changed to Dent's Bleach (10% H_2_O_2_, 20% DMSO in MeOH) for overnight at RT. Embryos were rehydrated using a descending MeOH row [75% MeOH, 50% MeOH and 25% MeOH in PBT (PBS+0.1% Tween)] for 20 min each at RT. 10% fetal calf serum (FCS) in PBDT (1% DMSO, 0.1% Tween in PBS) was used for the blocking buffer for 90 min. Primary MF20 (1:10 dilution in 1.5% FCS/PBDT) and S46 (1:50 dilution in 1.5% FCS/PBDT) antibodies were stained for overnight at 4 °C, respectively. Before and after primary and secondary antibody incubation, we washed embryos with 1.5% FCS/PBDT for 2 h. Hearts were dissected and mounted with VECTASHIELD® with DAPI and images were acquired using Confocal Microscopy.

### Aged zebrafish whole mount hematoxylin & eosin staining

Aged zebrafish were fixed in 4%PFA for 7 days at 4 °C and washed twice with PBS for 30 min. Fish were incubated in decalcification solution (20% EDTA pH 8.0 in DEPC treated water) for 10 days at RT and washed twice with DEPC treated water for 20 min. Fish were imbedded in Paraffin and sectioned. Sections were stained with Hematoxylin & Eosin and visualized by Zeiss Axioskop 2 plus (Carl Zeiss AG, Oberkochen, Germany).

### RNA extraction and quantitative real-time PCR

For the extraction of mRNA from *washc3^+/+^*, *washc3^+/−^*, and *washc3^−/−^* fish, 20–25 embryos collected in each group at 72 hpf, and adult hearts were harvested from fish and directly proceed to the RNA extraction. According to the provided protocol, we extracted mRNA with the RNeasy® Mini Kit (Qiagen, Hilden, Germany), and acquired mRNA were quantified using NANO drop 2000 (Thermo Fisher Scientific, Waltham, MA, USA). By using 1 µg of mRNA, cDNA was synthesized using Superscript III reverse transcriptase (Life Technologies, Carlsbad, CA, USA). Quantitative real-time PCR analysis using SYBR-Green master mix (Roche, Basel, Switzerland) according to protocols on a Light Cycler® 96 (Roche, Basel, Switzerland). Sequences of the used primers can be found in the Supplementary Tables S1.

### Birefringence analysis

Birefringence analysis was performed following a previous published protocol (20). Shorty, images were taken of embryos at 48, 72, 96, and 120 hpf under the Zeiss Axio Zoom V1.6 (Carl Zeiss AG, Oberkochen, Germany) microscope. Embryos were oriented in the 3% methylcellulose to prevent the movement while image taking. Birefringence intensity was quantified with ImageJ (version 1.53, NIH, Bethesda, MD, USA, https://imagej.nih.gov/ij/) by measuring mean gray value in selected skeletal muscle area.

### Quantitative assessment of embryonic cardiac function

Videos for assessing fractional shortening and heart rate were recorded using a Leica DM IL LED microscope (Leica Biostystems, Nussloch, Germany). Cardiac contractility of the embryos was evaluated by measuring the diastolic and systolic lumen areas at 48, 72, 96, and 120 hpf, as previously described (21).

### Touch evoked response assay

Based on a previously established protocol (20), to assess the responsiveness of zebrafish embryos to mechanical stimulation, embryos at 48, 72, and 120 hpf were gently stimulated using the tip of a fine needle. A prompt and directed escape response was considered indicative of normal reactivity. For quantitative analysis, the velocity and acceleration of the embryos were measured. Embryos were individually positioned at the center of a petri dish, stimulated with a needle, and their responses were recorded at 60 frames per second. Video recordings were subsequently analyzed using Tracker Video Analysis software (version 6.2.0; Open-Source Physics, Davidson College, USA; https://physlets.org/tracker/).

### Proteomic analysis

#### Sample preparation

Hearts were collected from 1-year-old adult zebrafish. Heart samples were homogenized using a tissue grinder (Cat# 947820B, Kisker Biotech, Germany) in 8 M Urea lysis buffer (8 M Urea, 50 mM Tris-HCL pH 8.0, 5 mM EDTA) and incubated on ice for 1 h. Samples were centrifuged at 20,000 g for 10 min. Cleared supernatants were subjected to determine protein concentration using Pierce™ BCA Protein Assay Kit (Thermo Fisher Scientific, Waltham, MA, USA). Each samples contained 40 μg of extracted protein and were precipitated using the Chloroform/Methanol method. Protein extracts were digested using the SP3 method with some modifications (22). Magnetic bead-based sample preparation was carried out using a prototype magnetic bead slurry (Promega, Cat. #916738, Madison, WI, USA). For each sample, 2 µL of Promega magnetic beads (100 mg/mL) were added and mixed with the protein solution by gentle pipetting. Protein binding was induced by the addition of 800 µL of 100% acetonitrile (ACN), resulting in a final concentration of 80% ACN. Samples were vortexed for 15–30 s to ensure homogeneity and incubated for 20 min at 24 °C with shaking at 1,000 g. Following incubation, samples were briefly centrifuged to collect liquid from the tube lid and then placed on a magnetic rack to separate the beads. The supernatant was removed and discarded. Bound proteins were washed three times with 1 mL of 80% ethanol and once with 1 mL of 80% ACN. Beads were collected on the magnetic rack, the wash solution was completely removed, and the beads were air-dried for 1 min. Beads were reconstituted in 100 µL of 100 mM TEAB pH 8.5. Trypsin was added in a ratio of 1:50, followed by overnight incubation at 37 °C. Samples were acidified using TFA to reach pH < 2. The pH of each sample was verified to be <2 before proceeding to StageTip desalting and LC-MS analysis. Digested proteins were desalted on self-assembled C18 Empore® extraction discs (3 M, Maplewood, MN, USA) (23).

#### LC-MS/MS analysis and data processing

Samples were suspended in 0.1% TFA and analyzed using an Ultimate 3,000 liquid chromatography system coupled to an Orbitrap QE HF (Thermo Fisher Scientific, Waltham, MA, USA) as described before (24). Briefly, peptides were separated in a 120 min linear gradient starting from 3% B and increasing to 23% B over 100 min and to 38% B over 20 min, followed by washout with 95% B. The mass spectrometer was operated in data-dependent acquisition mode, automatically switching between MS and MS2. MS spectra (m/z 400–1,600) were acquired in the Orbitrap at 60,000 (m/z 400) resolution and MS2 spectra were generated for up to 15 precursors with a normalized collision energy of 27 and an isolation width of 1.4 m/z. The MS/MS spectra were searched against the UniProt Danio rerio UP000814640 (August 2022), and a customized contaminant database using Proteome Discoverer 2.5 with Sequest HT (Thermo Fisher Scientific, Waltham, MA, USA). The fragment ion mass tolerance was set to 0.02 Da and the parent ion mass tolerance to 5 ppm. Trypsin was specified as an enzyme. The following variable modifications were allowed: Oxidation (M), Deamidation (N, Q), Acetylation (N-term), Met-loss (M), Met-loss + Acetyl (N-Term), whereas Carbamidomethylation (C) was set as a fixed modification. Peptide quantification was done using a precursor ion quantifier node with the Top N Average (*n* = 3) method set for protein abundance calculation.

### Data processing

Perseus software (version 2.0.11, Max Planck Institute of Biochemistry, Germany; https://maxquant.net/perseus/) was used for processing the result of proteomic analysis. All values were log2 transformed and filtered rows based on all three values identified at least one group. Missing values were replaced from a normal distribution for each replicate and differentially expressed proteins verified by ANOVA (Threshold, *p* *<* *0.05*). The mass spectrometry proteomics data have been deposited to the ProteomeXchange Consortium via the PRIDE partner repository with the dataset identifier PXD062550.

### Cell culture and adeno-associated virus transduction

AC16 cells (human ventricular cardiomyocytes) were cultured in DMEM/F12 GlutaMax medium (Thermo Fisher Scientific, Waltham, MA, USA) with 12.5% fetal calf serum (FCS) and 1% penicillin/streptomycin. Cells were maintained at 37 °C in a humidified incubator with 5% CO_2_. Culture medium was changed every 48 h and when cells reached confluence, cells were passaged using 0.05% trypsin and 0.02% EDTA. For the AAV virus transduction, we used 50,000 Multiplicity of Infection (MOI) and calculated volume of virus was mixed with serum and antibiotics free medium and treated for 24 h to transduce viruses into AC16. Medium with 6.5% FCS and 1% was changed and incubated at least 48 h for further experiments.

### Extraction of primary cardiomyocyte and seahorse assay

Isolation of primary cardiomyocyte from adult zebrafish was performed according to a previous established protocol (25). To obtain a sufficient number of cells for Seahorse assays, three to four hearts were collected per experimental group. The extracted primary cardiomyocytes, along with AAV-shRNA-transduced AC16 cells, were seeded into a Seahorse XF96 cell culture plate with appropriate medium in each well. Measurements were conducted following standardized protocols as previously described (26).

### Heart cryosection RNA scope

Heart dissected from aged zebrafish were fixed in 4% PFA with sucrose at RT for 1 h. After fixation, the hearts were washed in wash buffer (0.1 M phosphate buffer containing 4% sucrose) and incubated overnight at 4 °C in 0.1 M phosphate buffer containing 30% sucrose. The samples were then embedded in tissue freezing medium (Leica Biostystems, Nussloch, Germany), and cryosectioned at a thickness 10 μm using a cryotome (CM1950, Leica Biostystems, Nussloch, Germany). For RNA Scope analysis, sections were refixed with 4% PFA, washed with PBS, and subjected to a rehydration procedure using a graded ethanol series. hHydrogen peroxide and Protease IV were used for tissue digestion. Prewarmed probes were applied to sections and hybridize for 2 h at 40 °C. Hybridized sections were then incubated sequentially with AMP1 through AMP3 buffers for signal amplification, followed by incubation with RNAscope Multiplex FL V2 HRP and blocker for signal detection. All reagents were obtained from the RNAscope™ Wash Buffer Reagents kit, RNAscope™ H_2_O_2_ and Protease Reagents and RNAscope™ Multiplex Fluorescent Detection Reagents v2 (Advanced Cell Diagnostics, CA, USA).

### Statistical analysis

Statistical analysis was performed using GraphPad Prism 9 software. Mann–Whitney or unpaired *t*-test were used for the comparisons of 2 groups, and Kruskal–Wallis's test or one-way ANOVA were used for the comparisons of 3 groups depending on the result of the normality test. Parameters of all tests were two-tailed, statistical significance at *p* *<* *0.05* and results are represented as mean with standard deviation (SD) except OCR and ECAR curve displayed as mean with standard error of the mean (SEM).

## Results

### Knockdown of *washc3* induces neuromuscular defects and impairs heart function during early development in zebrafish

**Figure 1:**
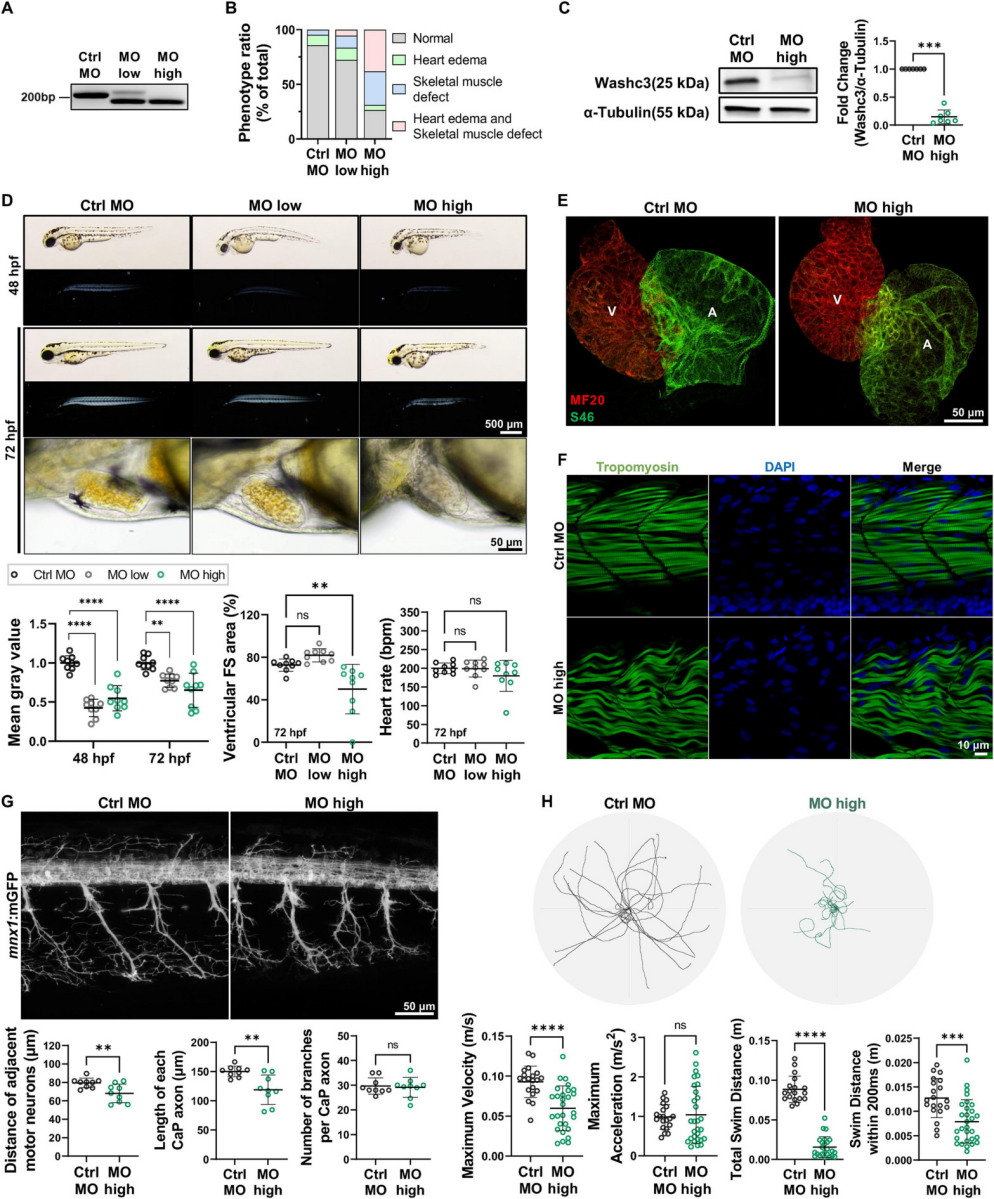
Knockdown of Washc3 in zebrafish embryo impairs neuromuscular development and cardiac function. **(A)** RT-PCR bands of *washc3* in control (ctrl) or *washc3* morpholino (MO) injected embryos at 72 h post-fertilization (hpf), validating MO against zebrafish *washc3* inducing deletion of 39 bp at the end of exon 2 via alternative splicing (MO-low: 250 μM of *washc3* MO; MO-high: 500 μM of *washc3* MO). **(B)** Phenotype ratios of ctrl or *washc3* morphants at 72 hpf (% of Normal, Heart edema, Skeletal muscle defect, Heart edema and skeletal muscle defect). **(C)** Immunoblots of Washc3 and α-Tubulin (a loading control) in ctrl or *washc3* morphants at 72 hpf. The blot intensity of Washc3 was normalized to α-Tubulin and fold change was calculated vs. ctrl morphants (SD, *n* = 7, Mann- Whitney test, ***p *<* *0.001* vs. Ctrl MO). **(D)** Lateral view of MO-injected embryos at 48 and 72 hpf in the images of brightfield (top panel) or birefringence (bottom panel) (scale bar: 500 µm). Magnified brightfield images of morphants' developing hearts showing unaffected cardiac phenotype by *washc3* MO at 72 hpf (scale bar: 50 µm). Quantified birefringence intensities, representing skeletal muscle development, were significantly decreased in *washc3* morphants at 48 and 72 hpf (SD, *n* = 9, Ordinary one-way ANOVA, **p *<* 0.005, ****p *<* *0.0001* vs. Ctrl MO). Heart function was assessed by measuring ventricular fractional shortening (FS) area (SD, *n* = 9, Ordinary one-way ANOVA, ns = not significant, **p *<* *0.005* vs. Ctrl MO) and heart rate (bpm: beats per minute) (SD, *n* = 9, Ordinary one-way ANOVA, ns = not significant vs. Ctrl MO). At 72 hpf, cardiac contractility was significantly reduced in *washc3* MO-high injected embryos. **(E)** Immunofluorescence (IF) staining of chamber-specific myosin heavy chain staining (MF20: ventricle and atrium, S46: atrium) for defining chamber specification and differentiation of morphants at 72 hpf (scale bar: 50 μm). **(F)** Confocal projections of morphants stained with Tropomyosin (green) and DAPI (blue) at 48 hpf (scale bar: 10 μm). **(G)** Representative confocal images visualizing the motor neuron in Tg(*mnx1*:mGFP) embryos injected with ctrl or *washc3* MO at 72 hpf. Statistical analyses revealed significantly decreased quantification of distance of adjacent motor neuron (SD, *n* = 9; Unpaired *t*-test, **p *<* *0.005* vs. Ctrl MO) and length of each CaP axon (SD, *n* = 9, Unpaired *t*-test, **p *<* *0.005* vs. Ctrl MO), but number of branches per CaP axon (SD, *n* = 9; ns = not significant vs. Ctrl MO) was not affected by MO-mediated knockdown of Washc3. **(H)** Overlaid swim paths of MO-injected embryos from touch evoked escape response assay demonstrates deteriorated motility of *washc3* morphants at 72 hpf. Statistical analyses of maximum velocity (m/s, SD; Ctrl MO, *n* = 20; MO-high, *n* = 28; Unpaired *t*-test, ****p *<* *0.0001* vs. Ctrl MO), maximum acceleration (m/s2, SD; Ctrl MO, *n* = 20; MO-high, *n* = 29; Mann–Whitney test, ns = not significant vs. Ctrl MO), total swim distance (m, SD; Ctrl MO, *n* = 18; MO-high, *n* = 24; Mann–Whitney test, ****p *<* *0.0001* vs. Ctrl MO), and swim distance within 200 ms (m, SD; Ctrl, *n* = 20; MO-high, *n* = 29; Unpaired *t*-test, ***p *<* *0.001* vs. Ctrl MO).

Given that knockdown of the WASH complex subunits Washc5 and Washc4 has previously been shown to impact skeletal muscle, cardiac, and neuronal development in zebrafish embryos (2, 9), we used morpholino (MO)-modified antisense oligonucleotides (MOs) to investigate the developmental role of Washc3 in zebrafish. To evaluate whether phenotypes scale with knockdown strength, we analyzed embryos injected with two morpholino doses (MO-low and MO-high). We injected wild-type embryos with a morpholino-modified antisense oligonucleotide (MO) targeting the splice donor site at the exon 2-intron 2 junction of the *washc3* gene. Knockdown efficiency and specificity were assessed by reverse transcription polymerase chain reaction (RT-PCR) followed by sequencing. Injection of 500 μM *washc3*-specific MO (MO-high) induced an aberrant splice variant lacking 39 base pairs at the end of exon 2, resulting in near-complete disruption of endogenous wild-type *washc3* transcript expression (Figure 1A, supplementary Figure S1). Western blot analysis confirmed a substantial reduction in Washc3 protein levels in *washc3* morphants at 72 h post-fertilization (hpf) (Figure 1C). Phenotypic characterization up to 72 hpf revealed that *washc3* morphants with high morpholino concentration exhibited reduced body length, axial curvature, and mild pericardial edema compared to control MO-injected and MO-low embryos (Figure 1B). To assess cardiac function, we measured ventricular fractional shortening (FS) and heart rate at 72 hpf. *Washc3* morphants displayed a significant reduction in FS, while heart rate remained unaffected (Figure 1D). Structural analysis of cardiac development using the myocardial markers MF20 (meromyosin/myosin heavy chain) and S46 (atrial myosin heavy chain) revealed no defects in cardiomyocyte specification or differentiation upon *washc3* knockdown (Figure 1E).

Based on prior findings involving other WASH complex subunits such as Washc4 and Washc5, we hypothesized that *washc3* knockdown may also impair neuromuscular development and embryo motility. First, skeletal muscle integrity was first evaluated using birefringence analysis. Birefringence intensity was significantly reduced in *washc3* morphants at 48 and 72 hpf, indicating severely disrupted myofibrillar organization. Whole-mount immunofluorescence of 48 hpf embryos stained for Tropomyosin confirmed aberrant muscle fiber architecture, with wavy and disrupted fibers observed in *washc3* morphants, as opposed to the parallel, tightly packed fibers seen in controls (Figure 1F). Next, we analyzed the effects of *washc3* knockdown on motor neuron morphology in the Tg(*mnx1*:mGFP) transgenic line (27), focusing on caudal primary (CaP) motor neurons to assess motor neuron development (28). *washc3* knockdown resulted in reduced intersomitic distance between adjacent motor neurons and shortened CaP axons, whereas axonal branching was preserved (Figure 1G). To functionally assess neuromuscular development, we performed a touch-evoked escape response assay at 72 hpf. *washc3* morphants demonstrated significantly reduced maximum swim velocity, total swim distance, and swim distance within the initial 200 ms post-stimulation, while maximum acceleration remained unchanged (Figure 1H). These results indicate impaired neuromuscular structure and function during early development.

Taken together, these data indicate that Washc3, like the WASH complex components Washc4 and Washc5, is critical for both cardiac and skeletal muscle development, further supporting the essential role of the WASH complex in orchestrating early vertebrate development.

### CRISPR/Cas9-mediated knockout of *washc3* causes transient striated muscle defects in zebrafish embryos

**Figure 2:**
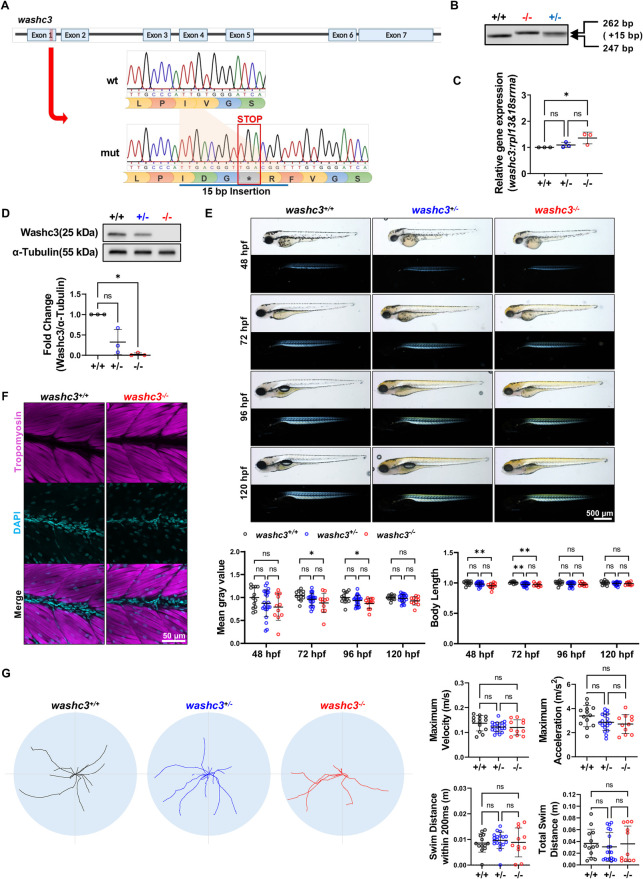
Stable *washc3* knockout zebrafish generated via CRISPR/Cas9 editing shows mild skeletal muscle growth phenotype and it is recovered by development. **(A)** Schematic showing CRISPR/Cas9-mediated gene editing of *washc3* exon 1. Sanger sequencing chromatograms are shown with inserted 15 bp causing premature stop codon. **(B)** RT-PCR analysis confirmed PCR products with or without mutation site in wild-type (wt) siblings (+/+, +/−) and *washc3* mutant (−/−). **(C)** qRT-PCR analysis of *washc3* in siblings and *washc3* mutant embryos at 72 hpf (SD, *n* = 3, Kruskal–Wallis test, *p *<* *0.05* vs. *washc3*^+/+^). **(D)** Immunoblots of Washc3 and α-Tubulin. The blot intensity of Washc3 normalized to α-Tubulin was calculated vs. *washc3*^+/+^ (SD; *n* = 3, Kruskal–Wallis test, *p *<* *0.05* vs. *washc3*^+/+^). **(E)** Lateral view of siblings and *washc3* mutants from 48 hpf to 120 hpf shown in brightfield images (top panel) and birefringence images (bottom panel, scale bar: 500 μm). Statistical analyses showing quantified mean gray value of birefringence intensity (SD; *washc3*^+/+^, *n* = 13; *washc3*^+/−^, *n* = 22; *washc3*^−/−^, *n* = 11; Ordinary one-way ANOVA, *p *<* *0.05* vs. *washc3*^+/+^) and body length (SD; *washc3*^+/+^, *n* = 13; *washc3*^+/−^, *n* = 22; *washc3*^−/−^, *n* = 11; Ordinary one-way ANOVA, **p *<* *0.005* vs. *washc3*^+/+^). Impaired skeletal muscle development of *washc3*^−/−^ at 72 hpf was alleviated by development (at 120 hpf). **(F)** IF images of *washc3*^+/+^ and *washc3*^−/−^ stained with Tropomyosin (magenta) and DAPI (cyan) at 96 hpf (scale bar: 50 μm). **(G)** Overlaid swim traces at 96 hpf from touch evoked escape response assay. Quantification of maximum velocity (m/s), maximum acceleration (m/s^2^), swim distance within 200 ms **(m)**, and total swim distance **(m)** didn't show significant difference between siblings and *washc3* mutant embryos (SD; *washc3*^+/+^, *n* = 13; *washc3*^+/−^, *n* = 19; *washc3*^−/−^, *n* = 11; Kruskal–Wallis test, ns = not significant vs. *washc3*^+/+^).

To elucidate the molecular mechanisms underlying Washc3-mediated regulation of cardiac and neuromuscular function, we generated a stable *washc3* knockout (KO) zebrafish line via CRISPR/Cas9 genome editing. As shown in Figure 2A, a 15-base pair insertion in exon 1 of the *washc3* gene introduced a premature stop codon, predicted to produce a truncated and non-functional Washc3 protein. Sequencing of genomic DNA from individual embryos confirmed the presence of this insertion by polymerase chain reaction (PCR) (Figure 2B). To determine the consequences of *washc3* deletion at both transcriptional and protein levels, we analyzed *washc3* mRNA and Washc3 protein expression in homozygous *washc3^−/−^* mutants. Quantitative real-time PCR (qRT-PCR) revealed an unexpected increase in *washc3* transcript levels, whereas Western blot analysis confirmed complete absence of Washc3 protein in *washc3^−/−^* embryos (Figures 2C,D), thereby validating the generation of a loss-of-function mutant suitable for subsequent downstream analyses.

We next sought to determine whether the phenotypic manifestations in *washc3^−/−^* mutants mirrored those observed in morphants. Representative birefringence images across developmental stages (48–120 hpf) are presented in Figure 2E. Consistent with the morphant phenotype, *washc3^−/−^* embryos displayed significantly reduced birefringence intensity between 72 and 96 hpf, indicative of impaired myofibrillar organization, along with reduced body length at earlier stages. However, these phenotypes progressively resolved, with skeletal muscle structure and body length indistinguishable from wild-type controls by 120 hpf. Cardiac function was similarly assessed in *washc3^−/−^* mutants. Compared to observations in morphants, no significant differences in heart rate and ventricular fractional shortening (FS) were detected during early development (Supplementary Figures S3A,B).

To assess whether early muscle abnormalities were fully resolved at later developmental stages, we performed Tropomyosin staining to examine muscle fiber architecture at 96 hpf. This analysis revealed no structural defects in *washc3^−/−^* embryo skeletal muscle (Figure 2F). Functional neuromuscular performance was evaluated using a touch-evoked escape response assay at 96 hpf, which showed no significant differences in locomotor function between *washc3^−/−^* embryos and their wild-type siblings (Figure 2G).

Taken together, these findings indicate that *washc3* knockout results in only mild and transient defects in skeletal muscle development, which are fully resolved by 120 hpf. This stands in stark contrast to the persistent early phenotypes observed in morphants, suggesting that redundant gene function or the influence of maternally deposited *washc3* transcripts may compensate for *washc3* loss-of-function during development.

### Aged *washc3* mutant zebrafish hearts lack pericardial membranes and exhibit epicardial remodeling

**Figure 3:**
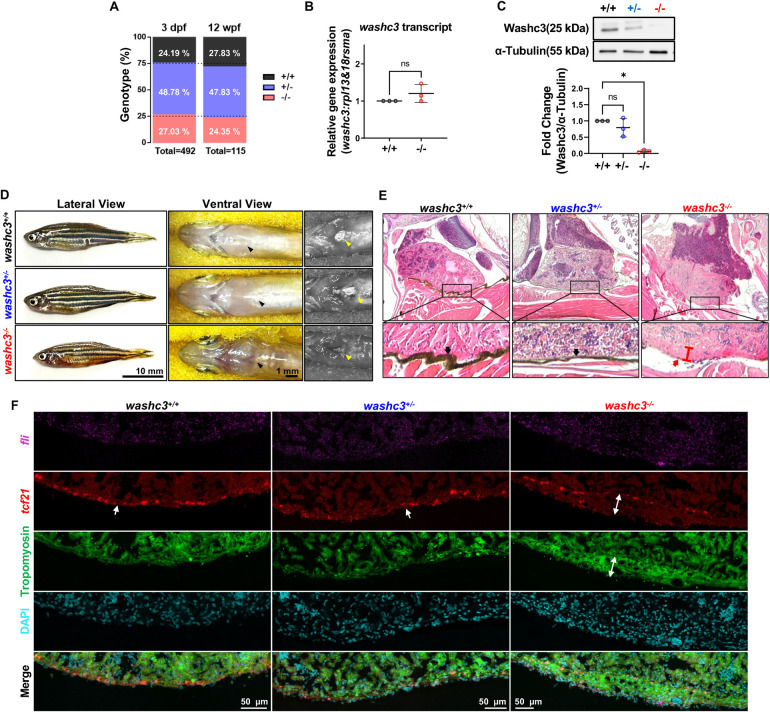
Aged Washc3 knockout zebrafish exhibits phenotypes of pericardial agenesis and compact outer layer of ventricle. **(A)** Relative ratio of offspring at 3 dpf and 12 weeks post fertilization (wpf) from *washc3*^+/−^ adult zebrafish (3 dpf: *washc3*^+/+^, *n* = 119; *washc3*^+/−^, *n* = 240; *washc3*^−/−^, *n* = 133; total *n* = 492; 12 wpf: *washc3*^+/+^, *n* = 32; *washc3*^+/−^, *n* = 55; *washc3*^−/−^, *n* = 28; total *n* = 115). **(B)** qRT-PCR analysis of *washc3* transcript at 1 year old adult zebrafish hearts (SD, *n* = 3, Mann–Whitney test, ns = not significant vs. *washc3*^+/+^). **(C)** Western blot analysis of Washc3 and α-Tubulin (SD, *n* = 3, Kruskal–Wallis test, *p *<* *0.05* vs. *washc3*^+/+^). Washc3 protein level was lost in the heart of 1 year old *washc3*^−/−^. **(D)** Representative lateral (left panel) and ventral (right panel) view of 17-months-old siblings (*washc3*^+/+^, *washc3*^+/−^) and *washc3*^−/−^ zebrafish. Black arrows on the ventral view marks the position of the heart before incision. Yellow arrows on the right panel of ventral view denotes pericardial membrane and its location after incision (Scale bar: Lateral view: 10 mm; Ventral view, 1 mm). **(E)** Hematoxylin and Eosin staining (H&E) staining of paraffin sections prepared from the whole mount aged zebrafish. Bottom panels are enlarged images from squared regions of top panels. Black arrows indicate pericardial membranes, and red arrow denotes outer layer of ventricle in *washc3*^−/−^. In the heart of *washc3*^−/−^, pericardial layer is not observed and outer layer of ventricle is thicker compared to siblings (*washc3*^+/+^, washc3^+/−^). **(G)** Confocal images of RNA scope staining with *fli* (endocardial cell: magenta) and *tcf21* (epicardial cell: red), as well as IF staining with Tropomyosin (myocardial cell: green) and DAPI (cyan) (scale bar: 50 μm). Outer layer of ventricle in *washc3*^−/−^ is thickened where t*cf21*^+^ cells are widely dispersed in the compact myocardium.

Following the resolution of skeletal muscle growth and organization defects by 120 hpf, *washc3* mutant zebrafish intriguingly displayed no overt phenotype during larval and juvenile, prompting further investigation into whether *washc3* deficiency might exert delayed or cumulative effects on viability, growth, and striated muscle homeostasis. To assess potential lethality associated with *washc3* loss, offspring from *washc3^+/−^* intercrosses were genotyped at two developmental stages: 3 days post-fertilization (dpf) and 12 weeks post-fertilization (wpf). Genotype distributions at both time points conformed to expected Mendelian ratios, indicating that *washc3* knockout does not result in embryonic or juvenile lethality in zebrafish (Figure 3A). To confirm the persistence of *washc3* knockout in adult animals prior to phenotypic analysis, we examined *washc3* transcript and protein levels in cardiac tissue from one-year-old zebrafish via qRT-PCR and Western blotting. Consistent with our embryonic data, *washc3^−/−^* hearts exhibited a nearly complete loss of Washc3 protein, while mRNA expression remained unaltered (Figures 3B,C), supporting post-transcriptional loss-of-function.

Strikingly, morphological abnormalities became evident in *washc3^−/−^* zebrafish by 17 months of age (Figure 3D). Ventral inspection revealed a conspicuous red and translucent tissue in the cardiac region (black arrow). Dissection and histological analysis of the heart exposed a complete absence of the pericardial membrane in mutants (yellow arrow), a finding confirmed by Hematoxylin and Eosin (H&E) staining of tissue sections. In wild-type zebrafish, a distinct brown-stained pericardial membrane (black arrow) separates the ventricle from the surrounding musculature; in contrast, this structure was absent in *washc3^−/−^* hearts, with the red arrow indicating its expected anatomical location (Figure 3E). In all aged mutants older than one year, the pericardial membrane was consistently absent, whereas embryos at 120 hpf displayed normal pericardial morphology (Figure 2E). These findings suggest that the pericardial membrane progressively degenerates and is ultimately lost during post-embryonic development.

To further determine how the cardiac architecture and pericardial membrane were affected in *washc3^−/−^* zebrafish, we performed high-resolution analysis of epicardial, myocardial, and endocardial layers using RNA scope with *tcf21-* and *fli-*specific probes—markers of epicardial and endocardial cells, respectively—alongside Tropomyosin immunostaining to visualize the myocardium. While no overt morphological differences were observed in the myocardium or endocardium between genotypes, the epicardium in *washc3^−/−^* hearts exhibited a markedly thickened and disorganized structure with two distinct layers of *tcf21*-positive foci (double arrows), in stark contrast to the thin, uniform monolayer observed in wild-type sibling hearts (arrowheads) (Figure 3F, supplementary Figure S4).

Collectively, these data reveal that while *washc3* is dispensable for zebrafish embryonic development, its loss is associated with progressive, age-associated structural cardiac abnormalities, most notably pericardial agenesis and epicardial remodeling.

### Comprehensive proteomic profiling identifies mitochondrial dysfunction and suppressed bioenergetic pathways in *washc3* mutant hearts

**Figure 4:**
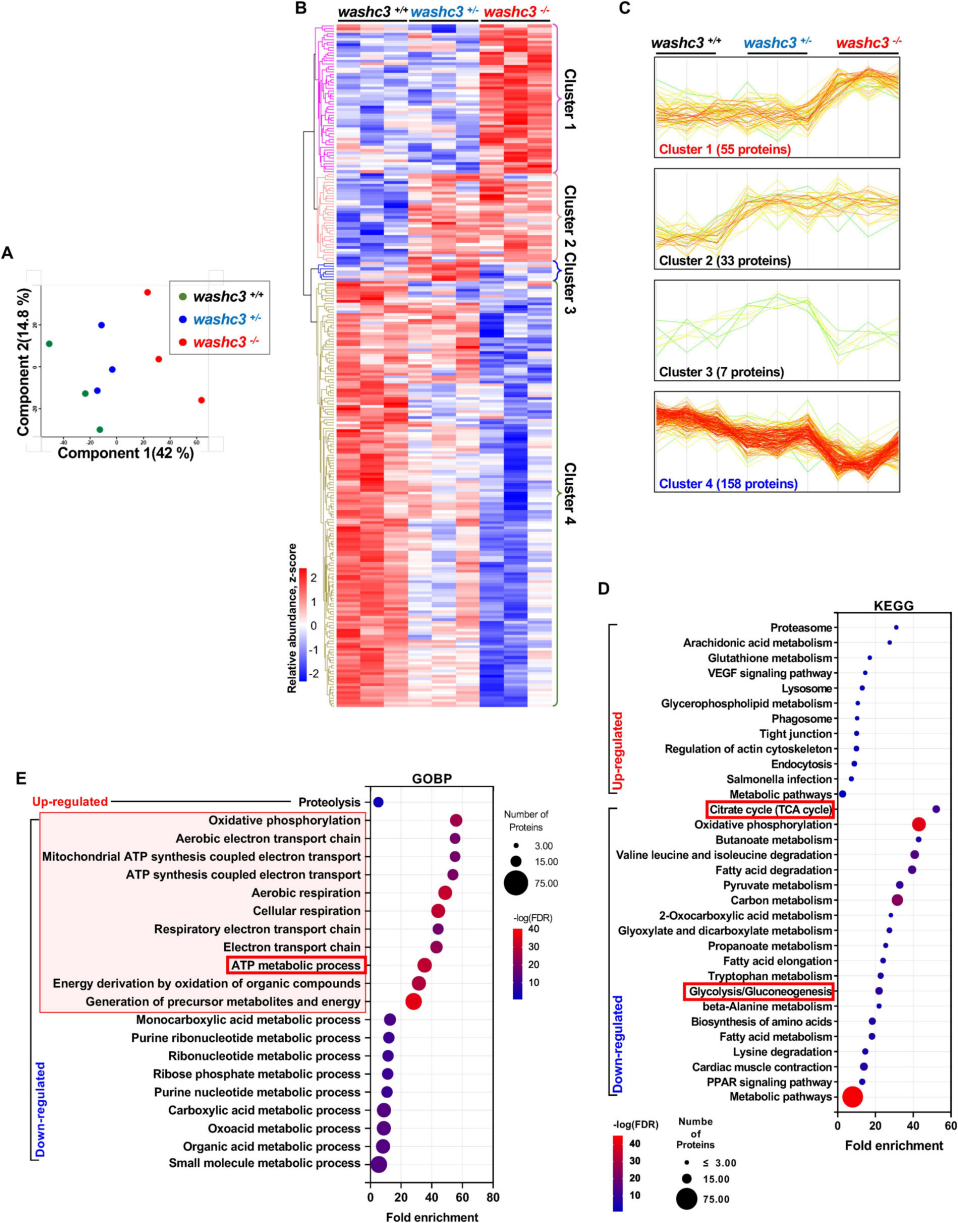
Proteomic analysis reveals downregulated mitochondrial proteins and ATP production related pathways in *washc3* knockout adult zebrafish heart. **(A)** Dot plot shows principal component analysis (PCA) of proteomics data from three biological replicates of heart-specific protein lysates in *washc3*^+/+^, *washc3*^+/−^, and *washc3*^−/−^ at 1 year old. **(B)** Unsupervised hierarchical clustering analysis (Heatmap) of 253 differently expressed protein. Each cluster was labelled by colors and numbers on the right side of the heatmap. **(C)** Representative images show distinct profiles of the clustered protein expression. Cluster 1 and 4 are highlighted by colors (red: up-regulated in *washc3*^−/−^, blue: down-regulated in *washc3*^−/−^) for the further analysis. **(D,E)** Gene ontology (GO) enrichment and KEGG pathway analysis of proteins clustered in **(B)** by ShinyGO 0.82 analyzer. Plots show pathways of GO terms Biological Process (BP) and KEGG pathways (cut off, *p < 0.05). The size of the circle represents the number of differently expressed proteins in each pathway category. The color of the circle indicates False discovery rate (FDR) as *p*-values and *X*-axis denotes Fold Enrichment.

To elucidate the functional role of Washc3 and its associated biological pathways in the zebrafish heart, we conducted quantitative proteomic profiling of cardiac tissue from three biological replicates per genotype. A total of 2,611 proteins were reliably identified and mapped to annotated zebrafish genes across all samples. Principal component analysis (PCA) revealed a distinct segregation of *washc3^−/−^* hearts from their wild-type and heterozygous counterparts along principal component 1, which accounted for 42% of the total variance (Figure 4A). To identify genotype-dependent proteomic alterations, we applied a two-way ANOVA to the normalized abundance values of all identified proteins, yielding 253 differentially expressed proteins (DEPs) at a significance threshold of *p* *<* *0.05*. Unsupervised hierarchical clustering of DEPs revealed four major clusters with distinct expression patterns (Figure 4B). Cluster 1 consisted of 55 proteins specifically upregulated in *washc3^−/−^* hearts, whereas cluster 2 encompassed 33 proteins elevated in both *washc3^−/−^* and *washc3^+/−^* samples. Cluster 3 included a smaller subset of seven proteins selectively upregulated in heterozygous hearts. Importantly, the majority of DEPs (158 proteins) were assigned to cluster 4, representing proteins significantly downregulated in *washc3^−/−^* hearts (Figure 4C).

To functionally characterize the affected pathways, we performed enrichment analyses on DEPs from clusters 1 and 4 using Kyoto Encyclopedia of Genes and Genomes (KEGG) and Gene Ontology (GO) annotations via the ShinyGO 0.82 platform. Enriched KEGG and GO Biological Process (GOBP) terms were ranked by fold enrichment and visualized in dot plots. KEGG pathway analysis showed that upregulated proteins were predominantly associated with pathways related to protein degradation and turnover, including the proteasome, lysosome, phagosome, and endocytosis. In contrast, proteins downregulated in *washc3^−/−^* hearts were significantly enriched for factors involved in metabolic processes, most notably those involved in mitochondrial energy production such as the citrate cycle (TCA), oxidative phosphorylation, pyruvate metabolism, fatty acid degradation, and glycolysis (Figure 4D). GOBP analysis further corroborated these findings, revealing that downregulated DEPs were primarily involved in ATP synthesis, aerobic electron transport, oxidative phosphorylation, and cellular respiration (Figure 4E). GO Cellular Component (GOCC) analysis localized these suppressed proteins to key mitochondrial structures, including the mitochondrial inner membrane, respiratory chain complexes, and the respirasome. Conversely, upregulated proteins were predominantly localized to the proteasome complex, consistent with an upregulation of protein degradation machinery (supplementary Figure S5).

In summary, these results indicate that Washc3 has a potential role in maintaining mitochondrial proteostasis and the integrity of oxidative energy metabolism in the zebrafish heart. The pronounced downregulation of mitochondrial proteins and associated metabolic pathways in *washc3^−/−^* mutants suggests impaired mitochondrial function, potentially triggering a compensatory upregulation of proteolytic systems. These findings underscore a critical role for Washc3 in cardiac energy homeostasis and protein quality control.

### Washc3 loss leads to impaired cardiac bioenergetics via coordinated suppression of mitochondrial gene and protein expression

**Figure 5:**
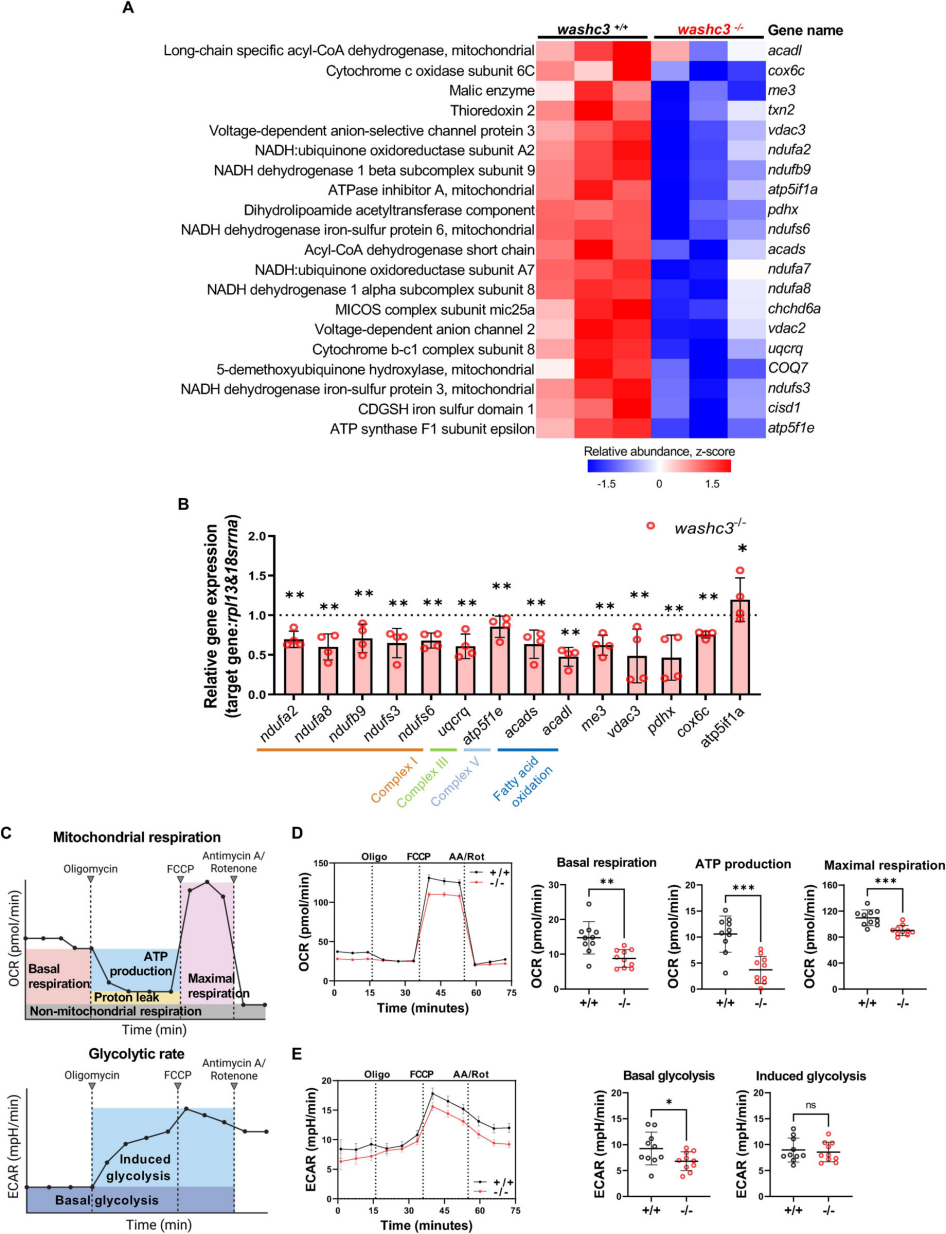
Loss of Washc3 results in suppression of mitochondrial proteins and mRNA leading to decreased mitochondrial respiration in adult zebrafish heart. **(A)** Heatmap of mitochondria related proteins significantly decreased in the *washc3* mutant hearts compared to *washc3*^+/+^ at 1 year old. **(B)** qRT-PCR analysis and relative expression of listed genes from **(A)** in adult zebrafish heart (SD, *n* = 4, Mann–Whitney test, **p *<* *0.005* vs. *washc3*^+/+^). **(C)** Descriptive illustration of seahorse assay (XF Mito Stress assay) (created by Biorender.com). Oxygen consumption rate (OCR) and extracellular acidification rate (ECAR) are measured before and after treatment of Oligomycin, FCCP, and AA/Rot. Each colored boxes represent the states of respiration possibly read out from the OCR or the ECAR curves. **(D,E)** OCR and ECAR curves in response to *washc3*^+/+^ and *washc3*^−/−^ primary cardiomyocytes dissociated from 1 year old adult zebrafish heart (SEM, *n* = 10). Corresponding quantifications of fundamental parameters shows significant decrease of basal respiration, ATP production, maximal respiration, basal glycolysis, and induced glycolysis in *washc3*^−/−^ primary cardiomyocytes (SD, *n* = 10 well per group, Mann–Whitney test, ns = not significant, * p *<* 0.05, **p *<* 0.005, ***p *<* *0.001* vs. *washc3*^+/+^ cardiomyocytes).

Next, we further investigated how this significant downregulation of pathways associated with mitochondrial function affected metabolic activity in zebrafish hearts. Proteomic analysis identified a predominant enrichment of DEPs related to mitochondrial function in the hearts of *washc3* mutant zebrafish. Figure 5A highlights the 20 most significantly downregulated mitochondrial-associated proteins, presented alongside their corresponding gene and protein annotations in a heatmap format. To investigate whether this downregulation occurs at the transcriptional level, we performed qRT-PCR using mRNA isolated from adult zebrafish hearts. The analysis revealed a consistent decrease in transcript levels a majority of the affected mitochondrial genes in *washc3^−/−^* hearts, corroborating the proteomic findings. Among the genes analyzed, transcript levels of components of the respiratory chain complexes and genes involved in fatty acid oxidation were reduced (Figure 5B).

While structural abnormalities were predominantly confined to the pericardium and epicardium in aged hearts, we hypothesized that disrupted mitochondrial energy metabolism could also play a critical role in compromising cardiomyocyte function within the myocardium. To evaluate the functional consequences of these molecular alterations, we isolated primary cardiomyocytes from *washc3^−/−^* and wild-type (*washc3^+/+^*) hearts and assessed mitochondrial respiration and glycolytic activity using the Seahorse XF96 Pro Analyzer (Figure 5C). Oxygen consumption rate (OCR) and extracellular acidification rate (ECAR) were measured in real time under basal conditions and following sequential treatment with mitochondrial inhibitors, including oligomycin (1 μM), FCCP (2 μM), and a combination of rotenone and antimycin A (1.5 μM). Cardiomyocytes from *washc3^−/−^* hearts exhibited significantly reduced basal respiration, ATP-linked respiration, and maximal respiratory capacity, alongside diminished basal glycolysis. However, glycolytic reserve, reflected by induced ECAR following mitochondrial inhibition, remained unaffected in mutants (Figures 5C–E).

Collectively, these results demonstrate that loss of Washc3 leads to a coordinated downregulation of mitochondrial transcripts and proteins, resulting in impaired oxidative phosphorylation and reduced cellular bioenergetic capacity in the adult zebrafish heart.

### AAV-mediated silencing of WASHC3 impairs mitochondrial respiration in human cardiomyocytes

**Figure 6:**
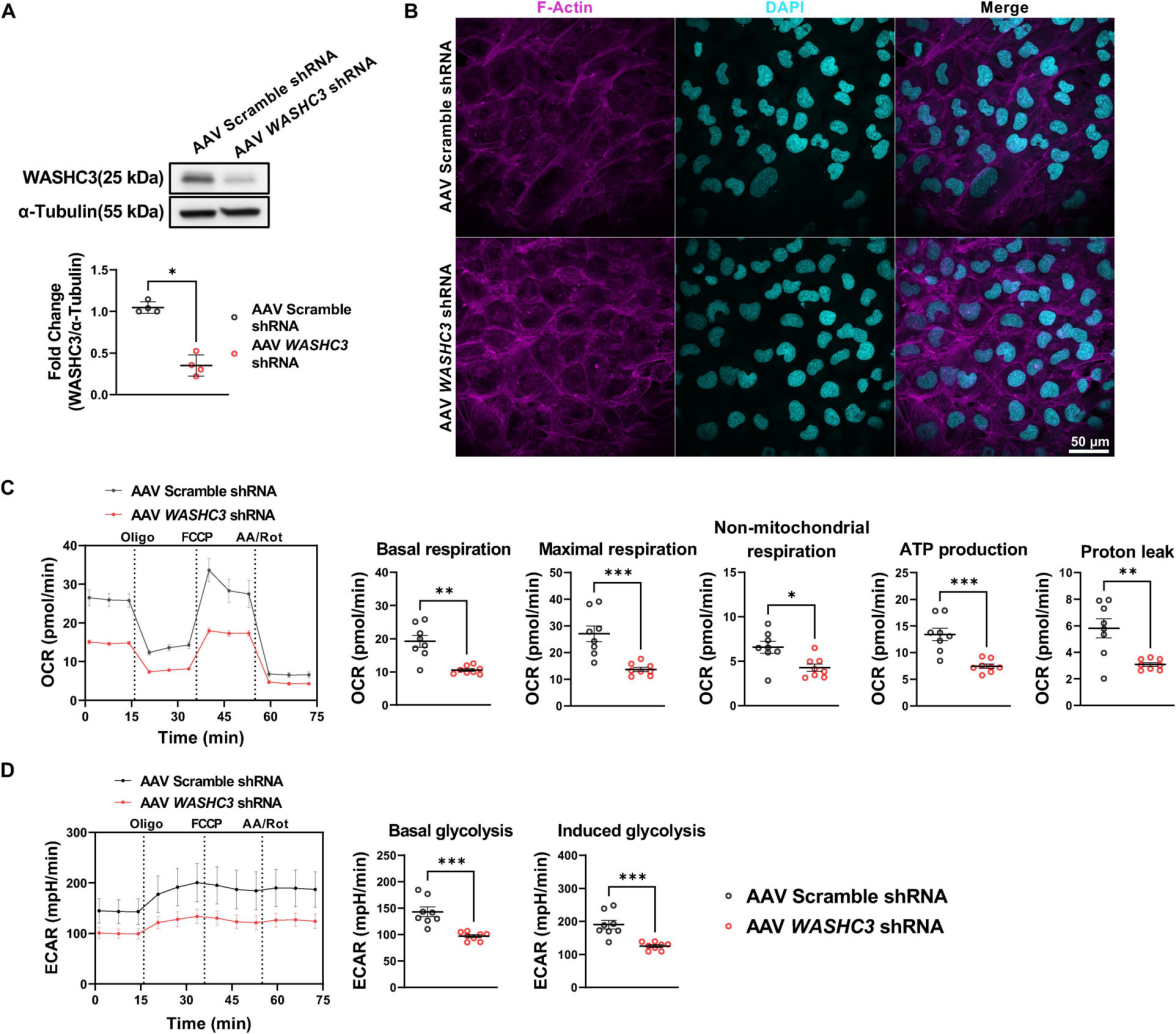
AAV-mediated knockdown of WASHC3 leads to defective mitochondrial respiration in human ventricular cardiomyocytes (AC16). **(A)** Immunoblot of WASHC3 and α-Tubulin (loading control) using AC16 cell lysate transduced with Adeno-associated virus (AAV) harboring scramble or *WASHC3* shRNA (SD, *n* = 4; Mann–Whitney test, *p *<* *0.05* vs. AAV6-Scramble-shRNA). **(B)** Confocal images of AC16 cells stained with F-Actin (magenta) and DAPI (cyan) at 72 h after transduction (scale bar: 50 μm). **(C,D)**) OCR and ECAR curves in AAV-scramble shRNA or -*WASHC3* shRNA transduced AC16 cells (mea*n* ± SEM, *n* = 10 well per group). Correspondent quantitative analyses of parameters reveal significantly repressed of mitochondrial respiration and glycolytic activity by AAV-mediated *WASHC3* knockdown. Basal respiration, Maximal respiration, non-mitochondrial respiration, ATP production, proton leak, basal glycolysis, and induced glycolysis were showed. (SD, *n* = 10 well per group of AC16 cells, Mann–Whitney test, *p *<* 0.05, **p *<* 0.005, ***p *<* *0.001* vs. Scramble shRNA).

To investigate whether the mitochondrial dysfunction observed in zebrafish hearts following *washc3* knockout is conserved in human cardiomyocytes, we employed an adeno-associated virus (AAV)-mediated approach using WASHC3-specific short hairpin RNA (shRNA). An initial serotype screening was performed with AAV1, AAV6, and AAV9 serotypes known to efficiently transduce muscle-derived cell lines at varying multiplicities of infection (MOI). Among these, AAV6 exhibited the highest transduction efficiency in the human AC16 cardiomyocyte cell line at 72 h post-transduction (Supplementary Figure S6). Effective silencing of WASHC3 was confirmed by immunoblotting, which showed a marked reduction in WASHC3 protein levels (Figure 6A). No overt morphological differences were observed between cells transduced with scrambled control shRNA and those with WASHC3-targeting shRNA, as assessed by confocal microscopy following F-Actin staining (Figure 6B). However, consistent with the phenotypes identified in *washc3*-deficient zebrafish cardiomyocytes, functional analysis using the Seahorse XF96 Pro Analyzer revealed significant mitochondrial impairment. Specifically, WASHC3 knockdown in AC16 cells led to a pronounced reduction in basal respiration, ATP production, proton leak, non-mitochondrial respiration, and maximal respiratory capacity (Figure 6C). Additionally, both basal and induced glycolytic activity, as indicated by extracellular acidification rate (ECAR), were significantly diminished in WASHC3-depleted cells (Figure 6D).

Taken together, these findings demonstrate that WASHC3 is important for maintaining both mitochondrial respiration and glycolysis in human cardiomyocytes, and underscore a potentially conserved role for WASHC3 in cardiac energy metabolism across species, from zebrafish to humans.

## Discussion

In this study, we uncover a previously uncharacterized role of WASHC3 in cardiac homeostasis by investigating its loss-of-function effects in zebrafish and human cardiomyocytes. While WASHC3 deficiency has been predominantly studied in the context of neurodevelopmental disorders, the present findings highlight a previously unappreciated role for WASHC3 in cardiac mitochondrial homeostasis and long-term cardiac maintenance. Although *washc3*-deficient zebrafish (*washc3^−/−^*) displayed no overt abnormalities during early development, aged mutants exhibited pronounced cardiac defects, including pericardial agenesis and thickening of the ventricular outer layer. Proteomic analysis of adult *washc3^−/−^* hearts revealed a significant downregulation of mitochondrial proteins, correlating with impaired mitochondrial respiration. These functional impairments were further corroborated in human AC16 cardiomyocytes following AAV-mediated WASHC3 knockdown, suggesting an unexpected conserved role for WASHC3 in mitochondrial homeostasis across species.

In this study, we demonstrate that antisense oligonucleotide-mediated knockdown of *washc3* in zebrafish embryos causes profound defects in embryonic heart function, skeletal muscle architecture, motor neuron development, and locomotor behavior, indicating a critical role for Washc3 in early neuromuscular and cardiac development. *washc3^−/−^* mutant embryos also showed early, mild signs of impaired growth and skeletal muscle development. The WASH complex, a well-characterized regulator of endosomal trafficking and protein homeostasis, has been implicated in human disease through pathogenic mutations in its subunits WASHC4 and WASHC5. A patient with a *WASHC4* mutation exhibited intellectual disability and developmental delay (5). Similar to this previously reported case, newly identified patients harboring a homozygous WASHC4 mutation presented with intellectual disability, craniofacial dysmorphism, and skeletal muscle abnormalities from early infancy (6). Mutations in WASHC5 have been associated with hereditary spastic paraplegia (HSP) type 8, a neurodegenerative disorder (7, 8). Furthermore, a *washc4* mutant mouse model exhibited disrupted endosomal and lysosomal homeostasis in the brain and showed severe cognitive impairment and motor deficits, recapitulating movement abnormalities observed in human patients (29). A recent study in humans showed that patients carrying mutations in WASHC3 present with variable neurodevelopmental disorders (4). However, the role of WASHC3 in cardiac development, structure, and function has not been investigated to date. Notably, the neuromuscular and cardiac phenotypes observed in *washc3*-deficient models may share a common underlying vulnerability related to impaired mitochondrial bioenergetics. Both developing skeletal muscle and cardiomyocytes are highly energy-demanding tissues, and disruption of mitochondrial protein homeostasis is expected to disproportionately affect their structural integrity and functional maturation. Thus, the early neuromuscular defects and the later-onset cardiac abnormalities described in this study can be interpreted within a unified framework of energy metabolism-dependent tissue maintenance.

Although *washc3*^−/−^ mutant zebrafish initially exhibited mild developmental abnormalities, these resolved during larval stages. In contrast, cardiac abnormalities became evident specifically in aged *washc3^−/−^* zebrafish (17 months old), including the absence of the pericardial membrane and a pronounced thickening of the epicardium. Pericardial agenesis is a rare congenital defect involving partial or complete absence of the pericardial sac. While complete agenesis is often asymptomatic, partial loss can result in serious complications such as cardiac displacement or vascular compression (30, 31). The mechanisms underlying pericardial agenesis are not fully understood, though defects in mesodermal differentiation during embryogenesis have been proposed (32). Interestingly, *washc3^−/−^* embryos exhibited normal pericardial formation until 120 hpf, while aged mutants lacked a pericardial membrane, suggesting that WASHC3 is required for post-developmental, age-associated maintenance of pericardial integrity rather than its initial formation. This raises new questions about the temporal and progressive role of WASHC3 in pericardial morphogenesis and warrants further investigation.

In zebrafish, the epicardium has been shown to play a pivotal role in cardiac development and regeneration following injury (33, 34). A recent study identified two distinct layers of *tcf21*-positive cells in the uninjured heart: an outer epithelial layer expressing *aldh1a2* and an inner mesenchymal layer lacking *aldh1a2* expression. The latter likely represents epicardial-derived progenitor cells undergoing epithelial-to-mesenchymal transition (EMT), contributing to cardiac repair (35). In the present study, we observed biphasic *tcf21* mRNA expression, with pronounced expression in the inner epicardial layer. This finding suggests that stress resulting from pericardial membrane degeneration may have activated regenerative mechanisms involving epicardial-derived progenitors.

The heart, as a highly energy-demanding organ, depends on mitochondrial oxidative phosphorylation for continuous ATP production (36, 37). The electron transport chain (ETC)—comprising complexes I-IV within the inner mitochondrial membrane—drives this process. Previous studies have shown that disruption of ETC components can result in severe developmental abnormalities, cardiac dysfunction, and increased mortality (38–40). Our proteomic analysis of adult *washc3^−/−^* hearts revealed downregulation of key components of the ETC, including NADH dehydrogenase (complex I), succinate dehydrogenase (complex II), cytochrome c reductase (complex III), cytochrome c oxidase (complex IV), and F-type ATPase. Importantly, these changes in mitochondrial protein abundance are directly supported by quantitative LC-MS/MS-based proteomic analysis, which provides primary protein-level evidence. qRT-PCR analyses were included as an independent orthogonal approach to assess whether these proteomic alterations are accompanied by corresponding changes at the transcript level.

Mitochondrial dysfunction is a hallmark of numerous diseases and can be triggered by mutations in both mitochondrial (mtDNA) and nuclear-encoded genes, ultimately disrupting ATP synthesis, elevating oxidative stress, and impairing cellular homeostasis (41). Beyond energy production, mitochondria are functionally integrated with endosomal trafficking pathways, which influence processes key to mitochondrial homeostasis such as including mitophagy and lipid/protein transport (42, 43). Recent evidence suggests that endosomal dysfunction can disturb mitochondrial quality control, emphasizing the potential for cross-talk between these organelles (44, 45). Several studies have highlighted the crosstalk between mitochondria, late endosomes, and lysosomes. For instance, the late endosomal-lysosomal membrane protein RAB7 has been shown to regulate mitochondrial fission and dynamics through direct mitochondria-lysosome contact sites (46). Moreover, in neurons, late endosomes transporting mRNAs encoding mitochondrial pro-survival proteins travel along axons, where local translation is facilitated at sites of endosome-mitochondria contact. These interactions are critical for maintaining neuronal viability (14). As the WASH complex is a master regulator of endosomal dynamics, its involvement in mitochondrial function represents a compelling area for future investigation, especially in metabolically active tissues such as the heart.

Our proteomic and functional analyses reveal a strong association between WASHC3 deficiency, reduced mitochondrial protein abundance, and impaired cardiac bioenergetics. To functionally validate our proteomic findings, we performed Seahorse XF mitochondrial stress assays on primary cardiomyocytes isolated from adult *washc3^−/−^* zebrafish. These assays revealed significant reductions in mitochondrial respiration and glycolytic capacity compared to wild-type controls. Importantly, similar defects were observed in human cardiomyocytes transduced with AAV containing WASHC3 shRNA, where both basal and maximal respiration, as well as glycolytic flux, were significantly impaired. While the present data identify WASHC3 as a previously unrecognized factor associated with the maintenance of mitochondrial bioenergetic capacity in cardiomyocytes, this study does not establish a direct molecular mechanism linking WASHC3 to mitochondrial regulation. Instead, our findings raise the possibility that WASHC3-dependent pathways, potentially involving endosomal or proteostatic processes, may influence mitochondrial homeostasis, a hypothesis that warrants further investigation. Although our experiments focused specifically on cardiomyocytes, WASHC3 is also expressed in various other tissues, including additional cardiac cell types such as epicardial cells, as well as neuronal tissues. To fully elucidate the broader physiological role of WASHC3, future studies should therefore extend these analyses to a wider range of cell types. In addition, based on our current findings, comprehensive assessments of cardiac function in adult and aged Washc3-deficient zebrafish will be an important next step in subsequent investigations.

Together, our results identify WASHC3 not only as a structural component of the WASH complex but also as a critical regulator of mitochondrial respiration. By integrating developmental, proteomic, transcript, and functional data from zebrafish and human cardiomyocytes, we propose that WASHC3 helps maintain mitochondrial homeostasis through regulation of electron transport chain components and energy metabolism pathways. These findings provide novel insights into the function of the WASH complex and open new avenues for investigating its role in mitochondrial regulation and cardiac physiology. They suggest that WASH complex components, beyond their established function in endosomal trafficking, may contribute directly or indirectly to mitochondrial homeostasis and energy metabolism in the heart. This expands the current understanding of the molecular pathways governing cardiac bioenergetics and highlights the potential relevance of WASH complex dysfunction in cardiometabolic disease.

## Data Availability

The datasets presented in this study can be found in online repositories. The names of the repository/repositories and accession number(s) can be found in the article/Supplementary Material.
