# Supplementary material for: WASHC3 knockout disrupts mitochondrial protein homeostasis and energy metabolism in cardiomyocytes
Source: Front Cardiovasc Med. 2026 Feb 12;13:1682381. doi: 10.3389/fcvm.2026.1682381 (PMC12936001; doi:10.3389/fcvm.2026.1682381)
Supplement: Supplementary file 1 [file Datasheet1.pdf]

S1 Fig

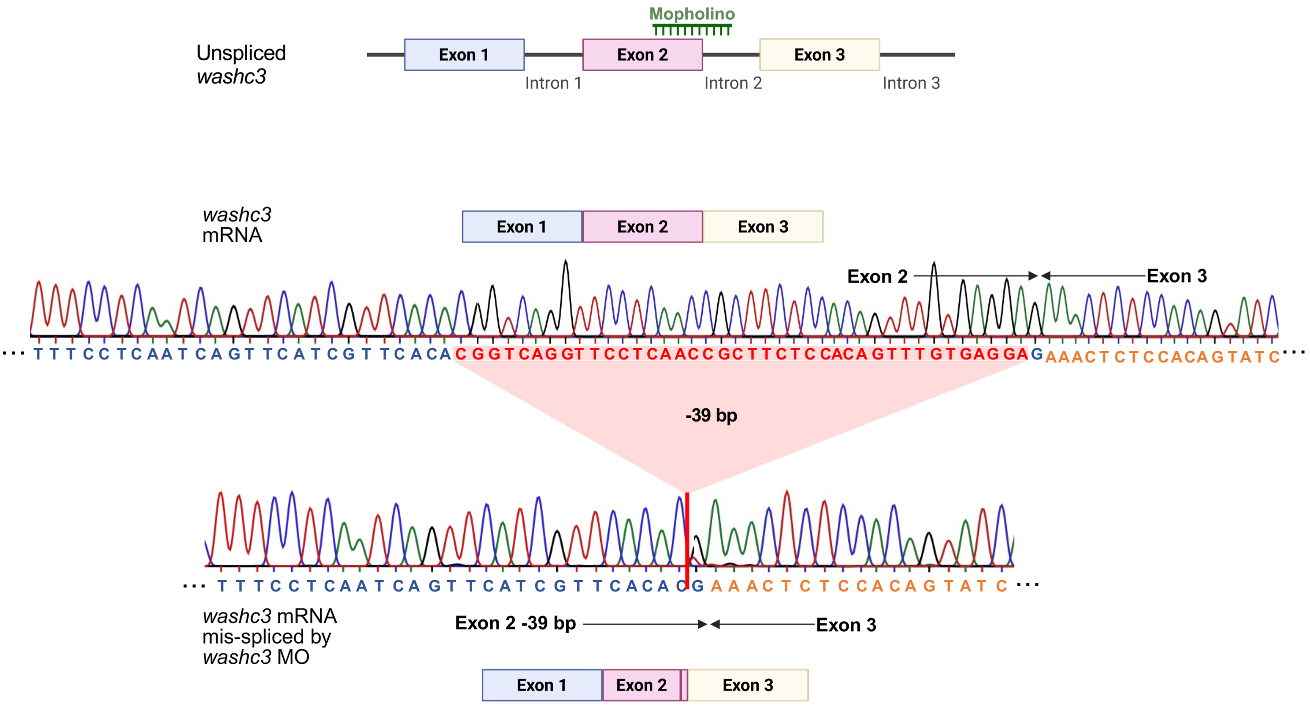

Mopholino
Unspliced
washc3
Exon 1
Exon 2
Exon 3
Intron 1
Intron 2
Intron 3
washc3
mRNA
Exon 1
Exon 2
Exon 3
Exon 2
Exon 3
··· TTTCCTCAATCAGTTCATCGTTCACACGGTCAGGTTCCTCAACCGCTTCTCCACAGTTTGTGAGGAGAAACTCTCCACAGTATC···
-39 bp
··· TTTCCTCAATCAGTTCATCGTTCACACGAAACTCTCCACAGTATC ···
washc3 mRNA
mis-spliced by
washc3 MO
Exon 2 -39 bp
Exon 3
Exon 1
Exon 2
Exon 3

S2 Fig

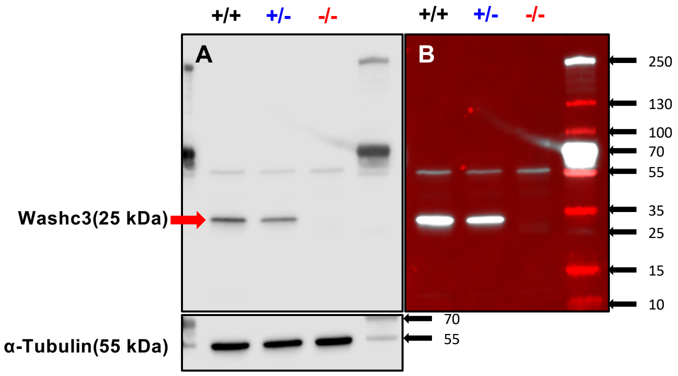
+/+
+/-
-/-
+/+
+/-
-/-
A
B
Washc3(25 kDa)
α-Tubulin(55 kDa)
250
130
100
70
55
35
25
15
10
70
55

S3 Fig

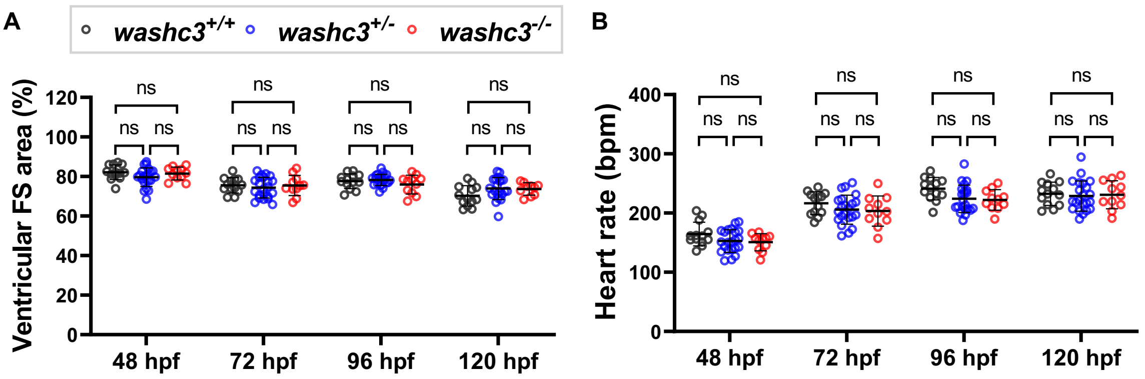
A
B
washc3+/+
washc3+/-
washc3-/-
Ventricular FS area (%)
Heart rate (bpm)
ns
48 hpf
72 hpf
96 hpf
120 hpf

S4 Fig

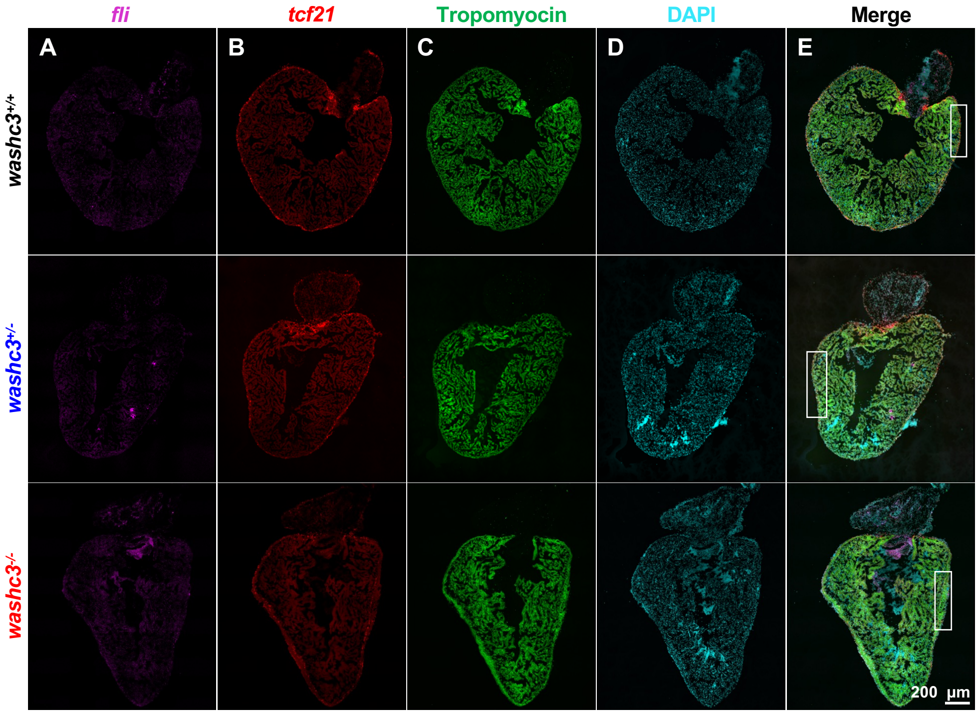

fli
tcf21
Tropomyocin
DAPI
Merge
A
B
C
D
E
washc3+/+
washc3+/-
washc3-/-
200 µm

S5 Fig

A

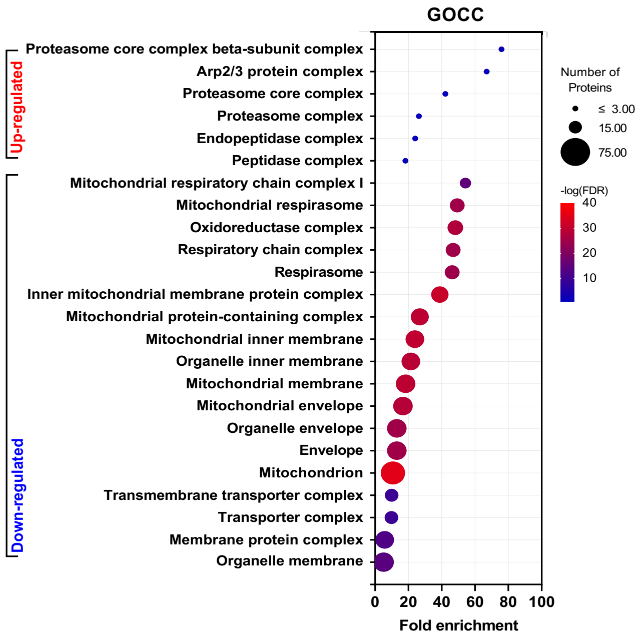
GOCC
Up-regulated
Down-regulated
Proteasome core complex beta-subunit complex
Arp2/3 protein complex
Proteasome core complex
Proteasome complex
Endopeptidase complex
Peptidase complex
Mitochondrial respiratory chain complex I
Mitochondrial respirasome
Oxidoreductase complex
Respiratory chain complex
Respirasome
Inner mitochondrial membrane protein complex
Mitochondrial protein-containing complex
Mitochondrial inner membrane
Organelle inner membrane
Mitochondrial membrane
Mitochondrial envelope
Organelle envelope
Envelope
Mitochondrion
Transmembrane transporter complex
Transporter complex
Membrane protein complex
Organelle membrane
0 20 40 60 80 100
Fold enrichment
Number of Proteins
≤ 3.00
15.00
75.00
-log(FDR)
40
30
20
10

S6 Fig

A

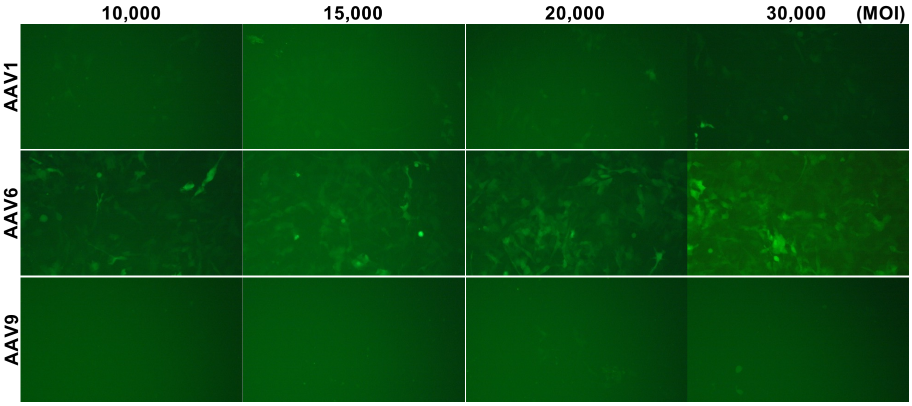
10,000
15,000
20,000
30,000
(MOI)
AAV1
AAV6
AAV9

B

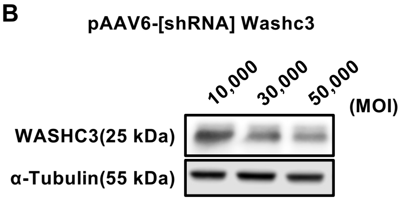
pAAV6-[shRNA] Washc3
10,000
30,000
50,000
(MOI)
WASHC3(25 kDa)
α-Tubulin(55 kDa)

S1 Table . Primers used in this paper

Genes		Sequence (5'-3')
washc3	Forward	GCAGAGGAGAATCGTGGCTT
	Reverse	ACTGTGGAGAAGCGGTTGAG
rpl13	Forward	TCTGGAGGACTGTAAGAGGTATGC
	Reverse	AGACGACAATCTTGAGAGCAG
18srrna	Forward	CACTTGTCCCTCTAAGAAGTTGCA
	Reverse	GGTTGATTCCGATAACGAACGA
ndufa2	Forward	GAATCTGCGTGAAATCCGCC
	Reverse	TCGGATCAGGATGGGGAACT
ndufa8	Forward	TGTGACAAGCCCAGCAAAGA
	Reverse	TGAAGGACTCAGCGCAGTTT
ndufa9	Forward	TTCTGGTCCAATCAGCACCC
	Reverse	CCAGTGATCGAGGCACCAAT
ndufs3	Forward	GTGGAGGTGCGCTATGATGA
	Reverse	GGTGCTTCTTTAGGCTCCCG
ndufs6	Forward	CCACACGGGACAGGTCTATG
	Reverse	CTCCATCACAGGACACCACC
uqcrq	Forward	TGGCTAAGGTGAGGCATGTG
	Reverse	GTCAGGTACGTCAGAGCCAG
atp5f1e	Forward	AGACATGTTTCCCGTGGGTC
	Reverse	GGCCTGGGTTAGAGAAGAGG
acads	Forward	GAGTTTGGGTGGAGCAGGAA
	Reverse	ACGATGACTCCTGTCGATGC
acadl	Forward	AAAGACAGCCGCTCATGGAA
	Reverse	AAAGCTTCAGCAGGCAAACG
me3	Forward	GGGGTATATTTGCCAGCGGA
	Reverse	GCAATAACTCCCAACGCGAC
vdac3	Forward	TCAGAACAACTTCGCCCTGG
	Reverse	AGCTGACCGTTCACCTTCTG
pdhx	Forward	CCCTCATCGCTCTGATGGTC
	Reverse	TCAAGCGCAGTTGTCTGAGT
cox6c	Forward	ATTTGCCCATTGCGTTTGCT
	Reverse	TACGCCTTTTTCCTGGGGTC
atp5if1a	Forward	GCTGGAGGATCATTCGGGAG
	Reverse	CGGATCTTGCCCTTGTGTCT

S1 Fig. Schematic representation of the morpholino target site and the resulting spliced mRNA product of *washc3*. A splice-blocking morpholino was designed to target the splice donor site at the junction of exon 2-intron 2. Morpholino interferes with normal splicing, resulting the removal of 39 nucleotides from the exon 2 after splicing. The 39 bp deletion was validate by Sanger sequencing and is highlighted in red.

S2 Fig. Western blot analysis of Washc3 protein levels in brain tissues of 1-year-old zebrafish. A) Original, unedited western blot images for Washc3 and α-Tubulin in brain tissue lysates from each indicated genotype. **B)** Blots highlighting the protein bands in white, with the molecular weight ladder indicated in red. The corresponding molecular weights of the ladder are shown on the right.

S3 Fig. Stable *washc3*$^{-/-}$ mutant showed no functional phenotype of heart during early development.

A-B) Assessment of heart function by measuring ventricular fractional shortening (FS) area (SD; *washc3*$^{+/+}$, n=13; *washc3*$^{+/-}$, n=22; *washc3*$^{-/-}$, n=11; Ordinary one-way ANOVA, ns=not significant vs. *washc3*$^{+/+}$) and heart rate (bpm: beats per minute, SD; *washc3*$^{+/+}$, n=13; *washc3*$^{+/-}$, n=22; *washc3*$^{-/-}$, n=11; Ordinary one-way ANOVA, ns=not significant vs. *washc3*$^{+/+}$) from wildtype siblings and washc3 mutants from 48 hpf to 120 hpf.

S4 Fig. Thickened epicardial layer observed in aged mutant hearts. Representative confocal microscopy images of whole-heart cryosections subjected to RNA scope and immunofluorescence staining for **A)** *fli,* **B)** *tcf21,* **C)** Tropomyosin, and **D)** DAPI (scale bar: 200 μm). **E)** The regions indicated by white squares in the merged images are enlarged in Fig. 3F.

S5 Fig. Proteomic analysis shows downregulated mitochondrial proteins in *washc3* knockout adult zebrafish heart. A) Gene ontology (GO) enrichment analysis of proteins clusters (shown in Fig. 4C) by ShinyGO 0.82 analyzer. Plots show pathways of GO terms Cellular Component (CC) (cut off, $*p<0.05$). The size of the circle represents the number of differently expressed proteins in each pathway category. The color of the circle indicates False discovery rate (FDR) as p-values and X-axis denotes Fold Enrichment.

S6 Fig. Comparison of transduction efficiencies of AAV serotypes in AC16. A) Representative images of AC16 cardiomyocytes transduced with AAV serotype 1, 6 and 9 at three different MOI after 72 hours post-transduction. **B)** Western blot showed efficiency of high MOI with *WASHC3* shRNA in AC16.
